# Extracellular vesicle-based Nanotherapeutics: Emerging frontiers in anti-inflammatory therapy

**DOI:** 10.7150/thno.47865

**Published:** 2020-07-09

**Authors:** Tao-Tao Tang, Bin Wang, Lin-Li Lv, Bi-Cheng Liu

**Affiliations:** Institute of Nephrology, Zhong Da Hospital, Southeast University School of Medicine, Nanjing, China.

**Keywords:** Extracellular vesicles, Anti-inflammatory therapy, Drug delivery, Inflammatory disease, Biotherapy

## Abstract

Dysregulated inflammation is a complicated pathological process involved in various diseases, and the treatment of inflammation-linked disorders currently represents an enormous global burden. Extracellular vesicles (EVs) are nanosized, lipid membrane-enclosed vesicles secreted by virtually all types of cells, which act as an important intercellular communicative medium. Considering their capacity to transfer bioactive substances, both unmodified and engineered EVs are increasingly being explored as potential therapeutic agents or therapeutic vehicles. Moreover, as the nature's own delivery tool, EVs possess many desirable advantages, such as stability, biocompatibility, low immunogenicity, low toxicity, and biological barrier permeability. The application of EV-based therapy to combat inflammation, though still in an early stage of development, has profound transformative potential. In this review, we highlight the recent progress in EV engineering for inflammation targeting and modulation, summarize their preclinical applications in the treatment of inflammatory disorders, and present our views on the anti-inflammatory applications of EV-based nanotherapeutics.

## Introduction

Inflammation underlies a wide range of physiological and pathological conditions 1-4. In infection or trauma, inflammation is essential to eradicate the noxious pathogens and heal the injured tissues to restore homeostasis. However, if the host fails to eliminate the initiating stimuli or generates a frustrated resolution response, the unresolved inflammation can cause organ dysfunction and become detrimental to the host. In such cases, chronic inflammation ensues and drives the development and progression of various noncommunicable diseases, such as cancer, cardiovascular disease and metabolic disease, which account for 70% of all deaths worldwide 5,6. Thus, considering inflammation as the cornerstone of many acute and chronic pathological processes, anti-inflammatory therapy is of paramount importance to improve patients' survival rate and survival quality.

With considerable progress in our understanding of the cellular and molecular mechanisms of inflammatory response, therapeutic agents varying from chemical compounds to gene drugs have been developed 7. However, their clinical translation is often limited by potential side effects, a slow onset of action, or a lack of efficacy, which might be attributed to the poor bioavailability, or to off-target effects after systemic administration 8,9. Therefore, advanced delivery strategies should be developed to improve pharmacokinetic properties and limit unwanted biological effects in order to realize the full translational potential of these potent drug candidates, particularly for gene and biologic therapies.

There has been growing interest in utilizing biologic nanocarriers by way of cell-derived membranous structures to develop innovative theranostics, and extracellular vesicles (EVs) present exciting potential opportunities and translational possibilities in this area because of distinct advantages such as low immunogenicity, biological barrier permeability, and intrinsic cell targeting properties 10-15. Based on the current knowledge of their size and biogenesis, EVs can be broadly divided into exosomes, microvesicles, and apoptotic bodies. Here, we focus on the first two classes: exosomes (30-150 nm in diameter) and microvesicles (50-1,000 nm in diameter), which originate from the endosomal system or are shed directly from the plasma membrane, respectively 16,17. Although EVs were initially regarded as cell dust with no biological significance, their implications in intercellular communication by transferring particular lipid, protein and nucleic acid contents between cells have been well documented recently 18-21. Importantly, their capacity to transfer cargos has introduced the possibility of using EVs as “Trojan horses” to deliver a variety of agents, including chemotherapeutics, miRNAs, siRNAs, proteins and even nanoparticles 10-12.

Diverging from existing reviews, we will focus on the current state of the art in manufacturing EVs as smart nanotherapeutics for anti-inflammatory therapy. We begin by taking stock of the latest advances in EV engineering technologies, with particular emphasis on their application in inflammation targeting and modulation. Then, we assemble emerging EV-based nanotherapeutics in the treatment of inflammatory diseases. Finally, we discuss the concerns and perspectives regarding the anti-inflammatory applications of EV-based nanotherapeutics.

## Engineering EVs as smart nanotherapeutics to target inflammation

Today, a broad spectrum of new drugs is being developed for the relief of inflammation. However, a sizable percentage of them fail to achieve the anticipated effect, in part because conventional therapies are not specific to the diseased tissue or because of the poor pharmacokinetics. EVs are highly tunable and can be engineered and customized for the targeted delivery of different kinds of cargo, which holds promise for reducing off-target effects, decreasing unwanted toxicities, and thereby enhancing a drug's therapeutic efficacy. Thus, employing EVs as a versatile drug carrier may advance and revive multitudinous therapeutic agents with inflammation-combating properties. This section will first consider the recent progress in the techniques of surface engineering and cargo loading, then discuss their applications in developing EV-based anti-inflammatory nanotherapeutics.

### Surface engineering of EVs to target inflammation

Surface engineering of EVs is an essential step for therapeutic applications because it endows EVs with additional properties, allowing them to accumulate at target sites, escape immunosurveillance, and achieve prolonged circulation time and enhanced intracellular uptake.

#### Surface engineering techniques

##### Cell engineering

Direct genetic engineering of the parental cell directly is a valid method for displaying encodable peptides, antibodies and proteins on the surface of EVs. This is performed either by inserting a targeting epitope into a protein domain, or by fusing a targeting sequence to the EV-enriched membrane proteins 22,23. Concerning the latter, the lysosomal protein lamp2b is widely used 24-27. For example, neuron targeting RVG peptide and α_v_ integrin-specific RGD (iRGD) peptide have been anchored on EVs through attachment to lamp2b for tissue-specific delivery 24,26. Despite these successes, it seems critical that peptides inserted to the N-terminus of lamp2b are probably degraded by endosomal proteases prior to surface expression 27. Alternatively, the targeting epitope can be fused to a desired protein domain, such as the transmembrane domain of platelet-derived growth factor receptor 28, glycosylphosphatidylinositol anchor peptides 29, or the C1C2-domain of lactadherin 30,31, instead of the whole proteins. However, it should be pointed out that these approaches may cause a functional loss of the host protein, and the recombinant construct may trigger immune activation.

Aside from improving targeting capacity, genetic engineering can be applied to increase the circulation time and cellular uptake of EVs. For example, the expression of vascular stomatitis virus-G on EV membranes enhanced their intracellular uptake by recipient cells 32. EVs engineered to overexpress CD47 exhibited superior escape from phagocytosis by the mononuclear phagocyte system 33.

##### Chemical modification

Click chemistry is a type of reaction commonly used for the bioconjugation of molecules to delivery systems via covalent bonds 34. In general, for EV modification, the alkynes are first grafted to the EV membrane and then reacted with azide-tagged molecules of interest via azide-alkyne cycloaddition. Therefore, this type of modification is less likely than others to dissociate away from the EV. Using this strategy, the azide-modified fluorophores 35, the integrin (ITG) α_v_β_3_-specific cRGD peptide 36, or the neuropilin-1-specific RGE peptide 37 have been successfully conjugated onto the EV surface. Of note, the critical alkyne modification may occur on the EV proteins rather than on the amines on membrane phospholipids. As such, the impacts of this modification on EV protein functions need to be comprehensively evaluated.

The hybridization of EVs with the synthetic liposomes is emerging as an alternative strategy to optimize and augment the properties of natural EVs. A freeze-thaw-induced 38 or PEG-induced 39 fusion of EVs and liposomes has been reported. With this strategy, the EV surface can easily be modified by fusion with liposomes embedding peptides or antibodies. Importantly, the hybrid EV/liposomes showed a better stability and half-life in circulation, as well as lower immunogenicity 38,39. Additionally, aptamers were displayed on EVs to realize a targeted antitumor delivery modality 40,41. Nanobody-PEG-lipid conjugates were inserted into the EV membrane to increase the target distribution and prolong the circulation time 42. Moreover, cationic lipids and pH-dependent fusogenic peptides were employed to increase the cellular EV uptake efficiency and cytosolic release of the cargo 43.

**Figure 1:**
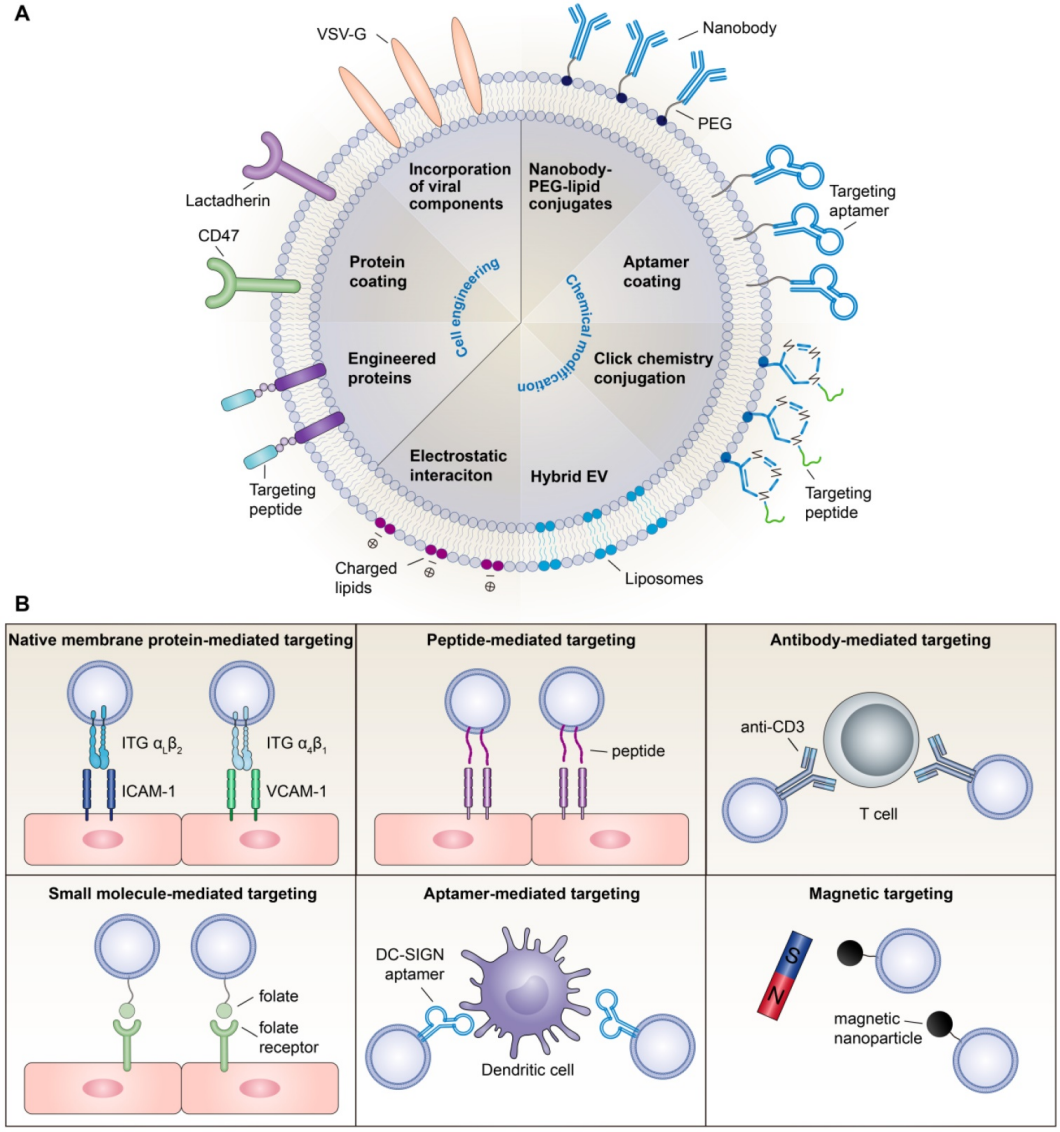
** Surface engineering of EV-based nanotherapeutics.** (**A**) Examples of surface functionalization strategies for EV-based nanotherapeutics. (**B**) Emerging strategies for developing inflammation targeting EV-based nanotherapeutics.

In summary, although surface engineering arms EVs with advanced functionality, research on these issues is still in its infancy. Modifications may alter the membrane protein structure, damage EV function, and exacerbate immunogenicity. Moreover, chemical routes require additional purification steps, which complicate the manufacturing process and increase the chance of EV loss. Thus, exploring more convenient, secure and effective functional moieties is necessary for broadening the therapeutic applications of EVs. Various EV functionalization approaches are shown in **Figure 1A**.

#### Emerging strategies for creating inflammation-targeting EVs

The homing of EVs towards certain recipient cells is mediated by natural membrane proteins or bioengineered moieties on the EV surface. For anti-inflammatory therapy, targeting EVs to inflammatory cells or inflamed tissues is the primary task.

##### Natural targeting capacity

There is mounting evidence that the cellular origin of EVs can guide their surface proteins, intrinsic targeting properties, and target cell tropism, so selecting the optimal pair of donor-recipient cells may promote specific EV uptake. For instance, T cell-derived EVs were efficiently taken up by macrophages and microglial cells 44, while EVs from mature dendritic cells were transferred unidirectionally to activated T cells 45. Recently, macrophage-derived EVs were shown to sense and migrate towards inflamed endothelial cells in both the kidney 46 and the brain 47, which was mediated by the interaction of ITG α_L_β_2_ and ITG α_4_β_1_ on EVs with intercellular cell adhesion molecule-1 (ICAM-1) and vascular cell adhesion molecule-1 (VCAM-1) on endothelial cells, respectively. Similarly, vesicles derived from activated neutrophils can selectively bind inflamed vasculature in the lung and brain because they possess intact targeting molecules of ITG β2 48-51. Inspired by the selective recruitment of leukocytes to the site of inflammation 52, leukocyte-derived EVs could facilitate inflammation-targeting drug delivery by the integrin patterns presented on the surface. Nevertheless, the integrin profile of these EVs needs to be further defined. Moreover, immune cell-derived EVs express abundant MHC class I and II molecules 53,54, which may increase the risk of immunogenicity; therefore, a long-term safety assessment is required.

##### Strategies for active targeting

In addition to native membrane proteins, EVs can be decorated with targeting ligands such as antibodies, peptides, small molecules and aptamers to achieve active targeting. In a recent study, Cheng et al. generated a novel synthetic multivalent antibodies retargeted exosome (SMART-Exo) through cell engineering, which expressed monoclonal antibodies specific for T cell CD3 and cancer cell-associated epidermal growth factor receptor (EGFR) and was shown to induce cross-linking of T cells and EGFR-expressing breast cancer cells 55. Using this strategy, antibodies for other immune cells or tissue cells are likely to be introduced onto the EV surface, allowing a more specific inflammation targeting. In parallel, targeting peptides have also been used to guide EVs towards lesion sites. For example, Tian et al. conjugated ITG α_v_β_3_-specific cRGD peptides to the exosomal surface by click chemistry, which efficiently targeted to the lesion region of the ischemic brain after intravenous administration 36. The ischemic myocardium-targeting peptide CSTSMLKAC (IMTP) 56 or cardiac-targeting peptide (CTP) 57 has also been displayed on EVs by fusion with lamp2 protein, conferring the capacity to target the heart. Other inflammation-targeting peptides, such as ICAM-1-binding Cyclo(1,12)PenITDGEATDSGC (cLABL) peptide 58,59 and VCAM-1-binding VHPKQHR peptide 60, have been screened; however, their targeting ability when conjugated to EVs remains to be confirmed.

Alternatively, small molecule-based targeting strategy has also been employed in EV-based therapy. For instance, folate, the most widely used small-molecule targeting agent 61, has been introduced to EVs to target cancer cells that express high levels of folate receptors 41. Considering that these receptors are also expressed on activated macrophages 62, folate-decorated EVs might be able to target macrophages to function in anti-inflammatory therapy. In addition, aptamers represent another low molecular weight targeting system. A comprehensive review of aptamer-guided EV theranostics in oncology is available elsewhere 63. With respect to inflammation targeting, a recent study successfully modified EVs with DNA aptamers against an adhesion receptor, ICAM-3 grabbing non-integrin (DC-SIGN), for the targeted delivery of therapeutic RNAs to dendritic cells 64. However, being nucleic acid-based, degradation in biological media is the most significant challenge of aptamer-targeted systems.

Besides targeting ligand-based strategies, magnetically and optically responsive hybrid-EVs were developed by incubating macrophages with iron oxide nanoparticles and photosensitizers, creating a method that uses magnetic targeting for the tissue-specific delivery of therapeutic EVs 65. However, magnetic nanoparticles may raise toxicity concerns, and there are difficulties in reaching deep tissues by this method. Overall, strategies for targeting inflammation currently remain limited. More active targeting methods with high affinity to tissue-specific cells need to be explored in the future (**Figure 1B**).

### Cargo engineering of EVs for anti-inflammatory therapy

EVs can be used as carriers for different therapeutic cargos, such as small molecule drugs, proteins, and genetic drugs. Here, the cargo-loading techniques and promising anti-inflammatory agents for EV-based nanotherapeutics will be discussed, with a focus on the improvement of the pharmacokinetics of these drugs delivered by EVs.

#### Cargo-loading techniques

##### Exogenous loading

**Figure 2:**
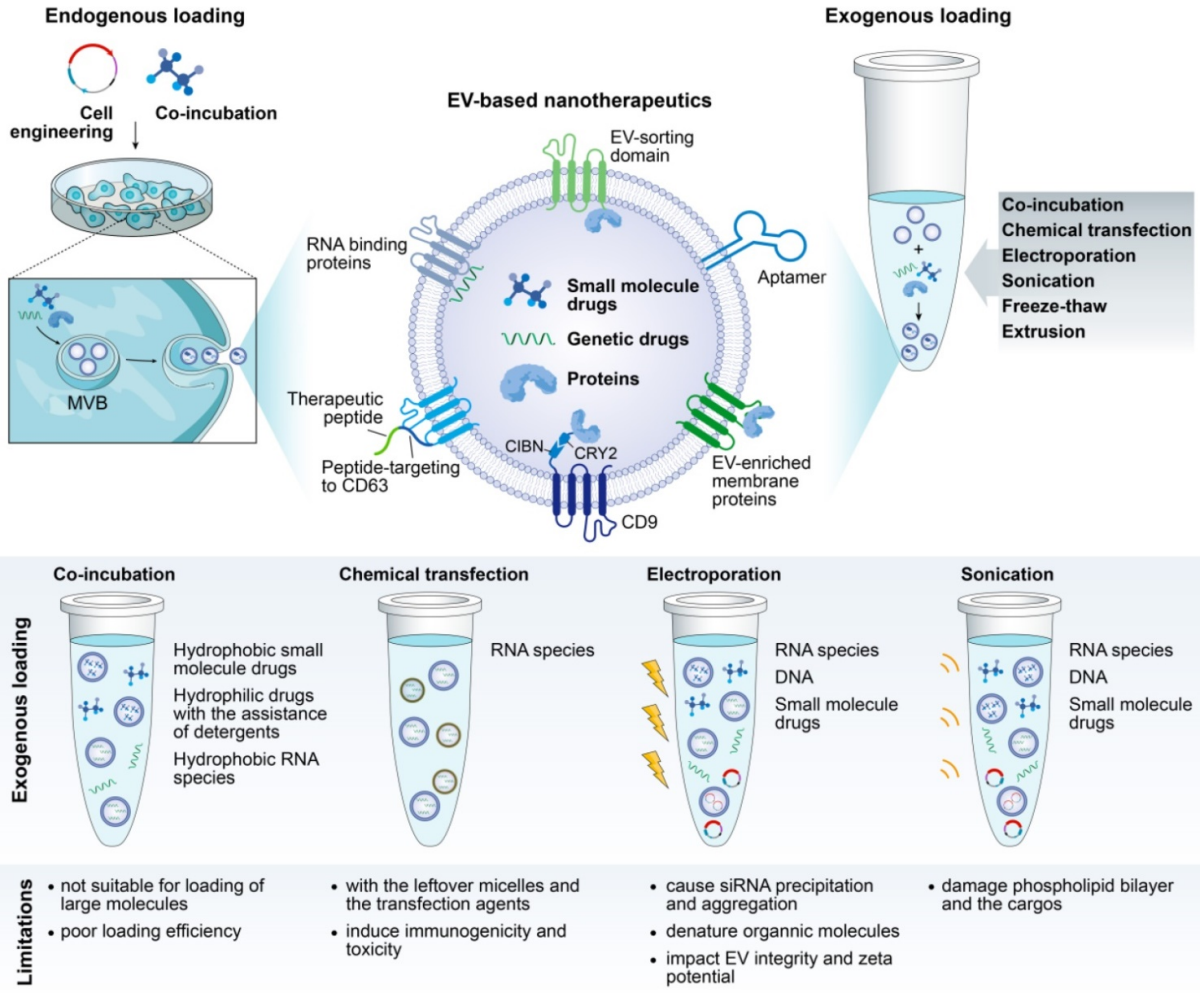
** Illustration of cargo-loading techniques to produce EV-based nanotherapeutics.** Cargo-loading can be carried out either endogenously (pre-loading parental cells with the cargo followed by inducing EV production) or exogenously (directly loading the EVs after their production and isolation).

Exogenous loading occurs after EV collection, with the desired therapeutic cargo packaged into EVs by simple coincubation or by chemical or physical approaches, each with its own advantages and limitations (**Figure 2**). Of note, violent loading procedures that may impact the EV integrity or cause immunogenicity or EV aggregation should be avoided.

Small hydrophobic molecules can be incorporated into EVs very efficiently by simple incubation 44,66. However, loading hydrophilic agents in this way requires the assistance of detergents, such as saponin, to loosen the vesicle bilayer 67. Interestingly, Gao et al. proposed a remote loading of hydrophilic drugs based on the pH gradient between the inside and outside of EVs. Preparing EVs in alkaline buffer to produce a pH gradient tripled the loading efficiency of piceatannol into EVs 49. Moreover, chemical transfection has been used to load siRNA in EVs by incubating EVs with siRNA-lipofectamine micelles 68. Despite the loading efficiency, EVs are difficult to purify from the surplus micelles and the transfection agents, which may elicit immunogenetic toxicity.

Physical approaches, such as electroporation, sonication and extrusion, are active loading strategies that generally involve breaking and then restoring EV membrane integrity to enable drug encapsulation. Of these, electroporation has emerged as a favorite method, particularly for RNA species packaging. For example, siRNA/shRNA has been electroporated into EVs to knock down oncogenic mutant KRAS in pancreatic cancer 33. However, poor loading efficiency has been reported in some cases, which may result from the formation of siRNA aggregates in the process of electroporation 69. A refinement of siRNA loading with hydrophobically modification was proposed, with convincing and efficient mRNA silencing of human antigen R 70 and huntingtin 71.

Recently, Gao et al. generated an anchor peptide, CP05, that targets CD63 on EVs, enabling direct and effective cargo loading on the surface of EVs. Painting EVs with CP05-conjugated muscle-targeting peptide in combination with CP05-conjugated phosphorodiamidate morpholino oligomer improved dystrophin expression and muscle function in a mouse model of muscular dystrophy 72.

##### Endogenous loading

Alternatively, the cargo can be endogenously loaded by incubating drugs of interest with parental cells directly or by genetically engineering the parental cells to overexpress a therapeutic RNA or protein, which will then be packaged into the EV cytosol or its membrane (**Figure 2**). However, how to ensure the selective sorting of desired drugs into EVs remains an open question. At present, a series of RNA binding proteins that specify the loading of miRNA and mRNA into EVs have been identified, including hnRNPA2B1 73,74, Y-box protein 1 75, SYNCRIP 76, and ELVA protein HuR 77, which are potential candidates for enabling the controlled loading of therapeutic RNA drugs. Intriguingly, an elegant tool (termed “EXPLORs”) that immobilizes proteins on the inner surface of EVs has been reported 78. In the EXPLOR producer cells, CRY2 was fused with a cargo protein (CRY2-cargo protein), and CIBN was fused to the N-terminus of an EV marker, CD9 (CIBN-CD9). As a result of the blue light irradiation, CRY2-conjugated cargo proteins were guided to CIBN domains on the surface of early endosomes, thereby effectively introducing cargo proteins into the newly generated EVs 78.

More recently, Yang et al. reported a cellular nanoporation (CNP) method for the scalable production of EVs, along with the loading of therapeutic mRNAs. When cells were transfected with plasmid DNAs by an electrical stimulus on the CNP silicon chip, large quantities of exosomes carrying transcribed mRNAs and targeting peptides were released, producing up to 50-fold more exosomes and an increase of more than 1,000-fold in exosomal mRNA transcripts compared to other strategies 79.

#### Promising anti-inflammatory agents for EV-based nanotherapeutics

##### Chemical therapeutics

The loading of small molecular weight drugs into EVs has been extensively studied, including anti-inflammatory drugs such as curcumin 44,66, piceatannol 49, aspirin 80, and dexamethasone 46. Curcumin is a plant-derived molecule with promising anti-inflammatory activity but suffers from poor solubility. Mixing curcumin with EVs allowed a large amount of drug loading into EVs. Importantly, EV packaging enhanced the solubility, stability and bioavailability of curcumin, which greatly improved its therapeutic efficacy in brain inflammation-related diseases 44,66. Likewise, dexamethasone is a widely prescribed medication used to treat inflammatory diseases; however, its clinical use is largely restricted due to adverse effects. A recent study prepared dexamethasone in EV formulations, conferring an increased capacity to reduce renal inflammation and fibrosis without significant adverse effects (e.g., hyperglycemia and suppression of HPA axis) during long-term use compared to the free drug 46. From these studies, EV-mediated delivery may open new opportunities for natural phytochemical compounds or drugs that have poor bioavailability, suboptimal pharmacokinetics, or off-target side effects.

##### Genetic therapeutics

Throughout the field, delivering nucleic acids, such as mRNA, miRNA and siRNA, within EVs has been widely favored, probably owing to their natural presence as EV cargo. For anti-inflammatory therapy, miRNAs have been successfully loaded into engineered or natural EVs. For example, exogenous miR-155 inhibitors were electroporated into B cell-derived EVs as a gene therapy strategy to relieve LPS-induced inflammation in macrophages. In contrast to conventional transfection methods, EV-mediated miRNA-155 inhibitor delivery resulted in functionally more efficient inhibition of tumor necrosis factor (TNF) production and less cellular toxicity 81. In addition, it should be noted that various miRNAs are able to modulate inflammation, but whether they are suitable for therapeutic application requires careful assessment. Manipulating miRNA expression may cause unintended biological effects, as the same miRNA can regulate multiple mRNA genes 82.

Unlike miRNA, siRNAs allow the facile and specific inhibition of any gene, and EV-mediated siRNA delivery is demonstrated to be an effective method for RNA interference (RNAi) therapy. To our knowledge, applying EV-siRNA in the fight against inflammation has not yet been reported, but many lessons can be learned from other fields, especially in the area of tumor RNAi therapy 83,84. First, using the transfection of leukemia cells as an example, EVs achieved remarkable delivery efficiency of antisense oligonucleotides without any toxicity to cells compared to commercial transfection reagents 85. Additionally, in T cells and monocytes, the gene mitogen-activated protein kinase 1 was selectively silenced by using siRNA-loaded EVs 86. Since most immune cells are difficult to transfect, the use of EVs as a vector can address this problem with enhanced transfection efficiency but low toxicity. Second, by the right of the modifiability of the EV membrane, EV-based RNAi drugs are more likely to reach the specific diseased tissues. For example, siRNA against BACE1 was encapsulated into EVs modified with neuron targeting RVG peptide, thereby leading to specific gene knockdown in the brain with minimal toxicity and immune stimulation, even during repeated systemic administration 24. Moreover, the surface expression of endogenous signaling ligands such as CD47 on EVs can increase the systemic circulation half-life and improve cellular uptake 33,87. Translating these advances into anti-inflammatory therapy may enable new breakthrough treatments. However, EV-based RNAi therapy still faces considerable challenges, including nonspecific gene silencing, limited silencing effects, and potential *in vivo* genotoxicity.

mRNA-based therapeutics hold great potential for the treatment of inflammation, as they can be exploited for precise and individualized therapy. A recent study demonstrated a proof of concept: implanting engineered producer cells termed EXOtic devices in living mice to generate therapeutic EVs loaded with biopharmaceutical-encoding mRNAs in-situ, which was intended to enhance EV production, specific mRNA packaging, and delivery of the mRNA into the target cells 88. In models of Parkinson's disease, EXOtic devices successfully ameliorated neurotoxicity and neuroinflammation by delivering catalase mRNA via EVs from implanted cells. Through this technology, we can load a specific mRNA of interest into EVs, and no need of concentration and purification of EVs as the traditional method does may further protect the stability of therapeutic mRNA inside the EVs.

##### Protein therapeutics

Proteins, such as enzymes, peptides, and cytokines, have become a class of important biotherapeutics for treating diseases because of their high biological activity and superb specificity 89. EVs have inherent advantages in delivering protein drugs with anti-inflammatory potency. For example, Kou et al. found that mesenchymal stem cells (MSCs) produce and secrete interleukin-1 receptor antagonist (IL-1RA), a natural inhibitor of the proinflammatory cytokine IL-1β, associated with EVs via the Fas/Fap-1/Cav-1 cascade triggered by TNF. IL-1RA-EV contributed to wound healing in both the gingiva and the skin 90. Another study engineered parental cells to release EVs overexpressing the anti-inflammatory cytokine IL-4 and containing the endogenous “eat me” signal lactadherin (Mfg-e8) on the surface to target phagocytes. A single injection of such EVs into the cisterna magna significantly ameliorated neuroinflammation by inducing the M2 phenotype in the recipient microglia 91. Recently, IκBα, a super-repressor of nuclear NF-κB activation, was loaded into EVs through the EXPLOR system to attenuate mortality and systemic inflammation in septic mouse models 92. These findings offer important inspirations that commandeering nature's own anti-inflammatory mechanisms to inhibit inflammation may accelerate the development of EV-based nanotherapeutics. Future therapies may be able to take advantage of other suppressors of inflammation, such as TGF-β and IL-10 family cytokines 93. Moreover, a kind of decoy EV was generated by presenting the TNF binding domain of human TNF receptor-1 on the EV surface, which antagonized TNF-induced signaling in cellular models of inflammation 94. In this way, decoy EVs displaying multiple receptors of inflammatory cytokines can be further exploited as biological sponges to absorb detrimental factors in blood or tissues.

Overall, EV-mediated drug delivery presents excellent application prospects in the anti-inflammatory therapy and gives new scope to a very large number of drugs by enhancing their solubility, bioavailability, stability, activity, or safety.

## Applying EV-based nanotherapeutics in the fight against inflammatory diseases

Two encompassing therapeutic applications of EVs can be summarized from current research. By virtue of their bioactive components, EVs have intrinsic therapeutic potential in tissue repair and regenerative medicine as well as in immunomodulation. On the other hand, natural or engineered EVs are being utilized as delivery vectors for different cargos of choice. Below, EV-based nanotherapeutics with anti-inflammatory properties and the underlying therapeutic mechanisms will be illustrated with contemporary examples taken from therapy for inflammatory diseases.

### EVs as therapeutic biomolecules with intrinsic anti-inflammatory activity

EVs from various sources have therapeutic potency, among which MSC-derived EVs appear particularly useful in the treatment of diverse conditions, including the treatment of inflammatory disorders of the respiratory system 95-98, heart 99-101, liver 102-104, kidney 105-108, nervous system 109-113, arthrosis 114-116, muscle 117-119 and others 120-124 (**Table 1**). The therapeutic action of MSC-EVs is reliant on their transfer of genetic materials and proteins. However, because most studies of MSC-EVs have focused on their efficacy rather than on their cargos, it is far from clear which anti-inflammatory entity is responsible for any given effect. A recent study delineated the components of IFNγ-activated MSC-derived EVs through deep RNA sequencing and proteomics, revealing that such EVs were rich in anti-inflammatory and neuroprotective RNAs and proteins 111. This study enhances the understanding of the potential mechanisms by which MSC-EVs exert therapeutic function in multiple sclerosis.

Concurrently, clinical trials have been initiated to assess the therapeutic value of MSC-EVs. The first documented clinical MSC-EV administration was performed on a steroid-refractory GvHD patient. The therapy reduced the pro-inflammatory cytokine response and attenuated the clinical GvHD symptoms, which were stable even after 4 months following MSC-EV treatment 125.

Subsequently, a randomized, placebo-controlled, phase 2/3 clinical pilot study showed that MSC-EVs safely and efficiently ameliorated the inflammatory immune reaction and improved overall kidney function in grade III-IV CKD patients 126. In addition, two ongoing trials are executed to use MSC-EVs for the treatment of type I diabetes mellitus (ClinicalTrials.gov identifier: NCT02138331) and macular holes (ClinicalTrials.gov identifier: NCT03437759). These clinical observations, together with evidence from preclinical studies, further indicate that native EVs from MSCs hold therapeutic promise in inflammatory diseases.

However, despite current successes, there are still some hurdles in the therapeutic use of MSC-EVs. First, the composition and function of EVs are susceptible to the producer cell state, and MSCs from different donors add another layer of uncertainty and risk. How to guarantee the stability and security of the MSC-EV therapy is a challenging problem. To address this, the underlying therapeutic mechanisms and pharmacodynamic effects of MSC-EVs should be defined. Second, owing to the lack of comparative studies, it is still unclear whether MSC-EVs work better than their parental cells. MSC-EVs protected against neonatal hyperoxic lung injury as effectively as MSC therapy 96, whereas other studies showed inferior potency of MSC-EVs compared to their parental cells for bone regeneration 127 and atherosclerosis 128. Furthermore, which subspecies of vesicles are responsible for the therapeutic efficacy remains controversial. A recent study suggested that MSC-derived exosomes were more efficient in suppressing inflammation in treating inflammatory arthritis than microvesicels 115. Considering the concurrence of pro- and anti-tumorigenic ability of MSC-EVs 129,130, further evaluation of their safety and potential in this regard is needed.

In addition to MSCs, EVs from other sources, such as amniotic epithelial cells 131, endothelial progenitor cells 132,133, and immune cells 134,135, show powerful effects in mitigating inflammation as well. For example, TNF-activated neutrophils released EVs displaying annexin A1 and/or phosphatidylserine on the surface, attenuating inflammation in the synovia by affecting macrophage polarization and potentially also fibroblast-like synoviocytes 136. Interestingly, microorganism-derived EVs are also considered to be a mine for pharmaceutical exploitation. *Akkermansia muciniphila* is a beneficial gastrointestinal microbiota whose EVs have recently been introduced to treat high-fat diet-induced obesity and show significant effects on adipose dysfunction, inflammation reduction, and obesity reversal 137.

### EVs as nanocarriers for anti-inflammatory therapeutics

#### Respiratory system inflammation

Acute respiratory distress syndrome (ARDS), a severe progression of acute lung injury (ALI), seriously threatens the lives of intensive care unit patients. However, there is no definitive pharmacotherapy in place for ARDS/ALI patients, in part because drugs cannot efficiently target inflamed lungs. Gao et al. reported a strategy that applied neutrophil-derived nitrogen cavitation (a physical force)-induced EVs to deliver the anti-inflammatory drug piceatannol (Pic-NC-EVs) for ALI treatment. As a result, Pic-NC-EVs significantly attenuated acute lung inflammation by inhibiting the NF-κB pathway and thus prolonged the mouse survival in sepsis 49. Moreover, Zhang et al. delivered anti-inflammatory small RNA molecules, such as Myd88 siRNA, miR-15a mimic, or miR-155 inhibitor, into lung macrophages respectively using serum-derived EVs as vehicles, successfully relieving LPS-induced lung inflammation 138.

Asthma is a common chronic respiratory disease characterized by airway remodeling and airway inflammation. Anti-inflammatory and anti-airway remodeling therapies are the two basic treatments. Recently, Shang et al. modified MSCs to generate EVs enriched with the circular RNA-mmu_circ_0001359, which enhanced FoxO1 signaling-mediated M2-like macrophage activation via sponging miR-183-5p and thereby suppressing pro-inflammatory cytokine expression and attenuating airway remodeling 139. The findings above highlight the promise of EV-based nanotherapeutics for lung inflammation. Furthermore, compared to artificial nanoparticles, EVs may not induce cytotoxicity, inflammation, changes in airway resistance or structural damage of the lung tissue.

#### Cardiovascular disease

Cardiovascular disease (CVD) is the leading cause of morbidity and mortality worldwide. Inflammatory processes are firmly established as central to the development and complications of CVD and have become a target of therapy. Increasing evidence has shown that EV-based therapy represents a promising tool to combat inflammation in CVD, such as myocardial infarction, stroke, pulmonary hypertension and others 140. For example, localized injection of miRNA-181a- overexpressing MSC-EVs inhibited the inflammatory response and raised the Treg cell ratio through inhibition of c-Fos protein, encouraging restoration of the infarcted hearts 141. However, the injected EVs may be eliminated quickly due to a lack of support, since they were undetectable 3 h after myocardial injection 142. To prevent this rapid elimination, MSC-EVs were encapsulated in functional peptide hydrogels to prolong EV retention in the heart, which resulted in better cardiac function with reduced apoptosis, inflammation and fibrosis after myocardial infarction when compared with free EVs 143. Of note, it remains to be established whether the systemic delivery of EVs would trigger similar positive effects because circulating EVs may be rapidly cleared by the mononuclear phagocyte system, and the continuous shear stress from constant blood flow may prevent nontargeted EVs from accumulating in the heart.

#### Neuroinflammation

EV-based nanotherapeutics have been widely applied in the treatment of nervous system diseases, such as Alzheimer's disease 24, Huntington's disease 71,144, Parkinson's disease 145-148, and ischemic reperfusion injury 149,150. For example, Parkinson's disease is a neurodegenerative disorder marked by the elevation of α-synuclein, brain inflammation, and reactive oxygen species. Haney et al. harnessed EV therapy for the intranasal delivery of catalase to the brain, reducing brain oxidative stress and inflammation and improving neuronal survival in a mouse model of Parkinson's disease 145. Kalani et al. proved the neuroprotective effects, such as reduced inflammation, restored blood brain barrier and overall improved neurological score, of curcumin-loaded embryonic stem cell-derived EVs in an ischemia-reperfusion injury model 149. Dong et al. reported that neutrophil membrane-derived vesicles can specifically target inflamed brain endothelium during ischemia-reperfusion injury and deliver RvD2 to prevent neuroinflammation 50. Importantly, these studies suggest that the nanosize, cellular origin, and other properties made EVs capable of passing through the blood brain barrier, thus highlighting the potential of EV-based nanotherapeutics for the treatment of central nervous system disorders.

#### Autoimmune diseases

Autoimmune diseases refer to a group of chronic inflammatory diseases caused by self-immune responses to autoantigens; such conditions include rheumatoid arthritis, systemic lupus erythematosus, type 1 diabetes, and multiple sclerosis, among others. The current conventional therapies, notably corticosteroids and immunosuppressors, seriously endanger human health. To solve this dilemma, EV-based nanotherapeutics have been tested, and certain results have been achieved in treating rheumatoid arthritis 151 and multiple sclerosis 44,91,152. Chen et al. transfected MSCs with a miR-150-5p expression plasmid to harvest miR-150-5p-enriched EVs, which significantly reduced hind paw thickness and clinical arthritic scores in collagen-induced arthritis mice by inhibiting synoviocyte hyperplasia and angiogenesis 151. Yu et al. generated therapeutic EVs from modified dendritic cells expressing membrane‐associated TGF‐β1 (mTGF‐β1). The mTGF‐β1-EVs possessed powerful immunosuppressive ability and effectively inhibited the development and progression of multiple sclerosis in different strains of mice 152. Moreover, the aforementioned EVs loaded with the anti-inflammatory drug curcumin 44 or IL-4 protein 91 have also been proven to reduce clinical signs in a mouse model of multiple sclerosis.

#### Renal inflammation

The role of EVs in the pathogenesis of renal inflammation is widely accepted 153-155, but their therapeutic application has just begun. In the first study, MSC-EVs were used to deliver miR-let7c to damaged kidneys, achieving the repression of renal fibrosis by down-regulating TGF-β1 protein expression in UUO mice 156. A subsequent study employed macrophage-derived EVs for the targeted delivery of dexamethasone to the inflamed endothelium, not only improving the anti-inflammatory efficacy but also reducing the side effects of dexamethasone. Interestingly, in addition to the drug, glucocorticoid receptors in the macrophages were also packaged and delivered to renal cells, thereby increasing cellular levels of the receptor and improving cell sensitivity to dexamethasone 46. Considering that the downregulation of glucocorticoid receptors is associated with acquired resistance 157, such EVs loaded with the drug and adjunct receptors may be beneficial for steroid-resistant patients. Furthermore, these findings provide some preliminary evidence that in the context of kidney injury, EVs can cross the glomerular filtration barrier by right of their small size and good biocompatibility; however, additional discussion and analyses are needed to clarify this issue.

#### Other diseases

Inflammatory bowel disease, including Crohn's disease and ulcerative colitis, is a chronic and relapsing inflammatory condition of the gastrointestinal tract. Current medical management strategies cannot keep patients in remission for the long term or may cause serious side effects due to the typically high dosage required. Recently, the well-known anti-inflammatory miR-146a was packaged into MSC-EVs for the treatment of experimental colitis, leading to inhibition of the NF-κB-dependent activation of inflammatory gene transcription by targeting TNF receptor-associated factor 6 and IL-1 receptor-associated kinase 1 in colon tissue 158.

Moreover, EV-based nanotherapeutics have also been applied in a murine model of duchenne muscular dystrophy 72,159, a fatal neuromuscular disorder that manifests as progressive muscle wasting. For instance, myostatin propeptide, which is a natural inhibitor of mature myostatin, was anchored to the surface of NIH3T3 mouse embryonic fibroblast cell-derived EVs by fusing the inhibitory domain of myostatin propeptide into the extracellular loop of CD63, which enhanced the serum stability and delivery efficiency of the propeptide and thereby led to a significant increase in muscle mass and functional rescue in *mdx* mice 159.

**Figure 3:**
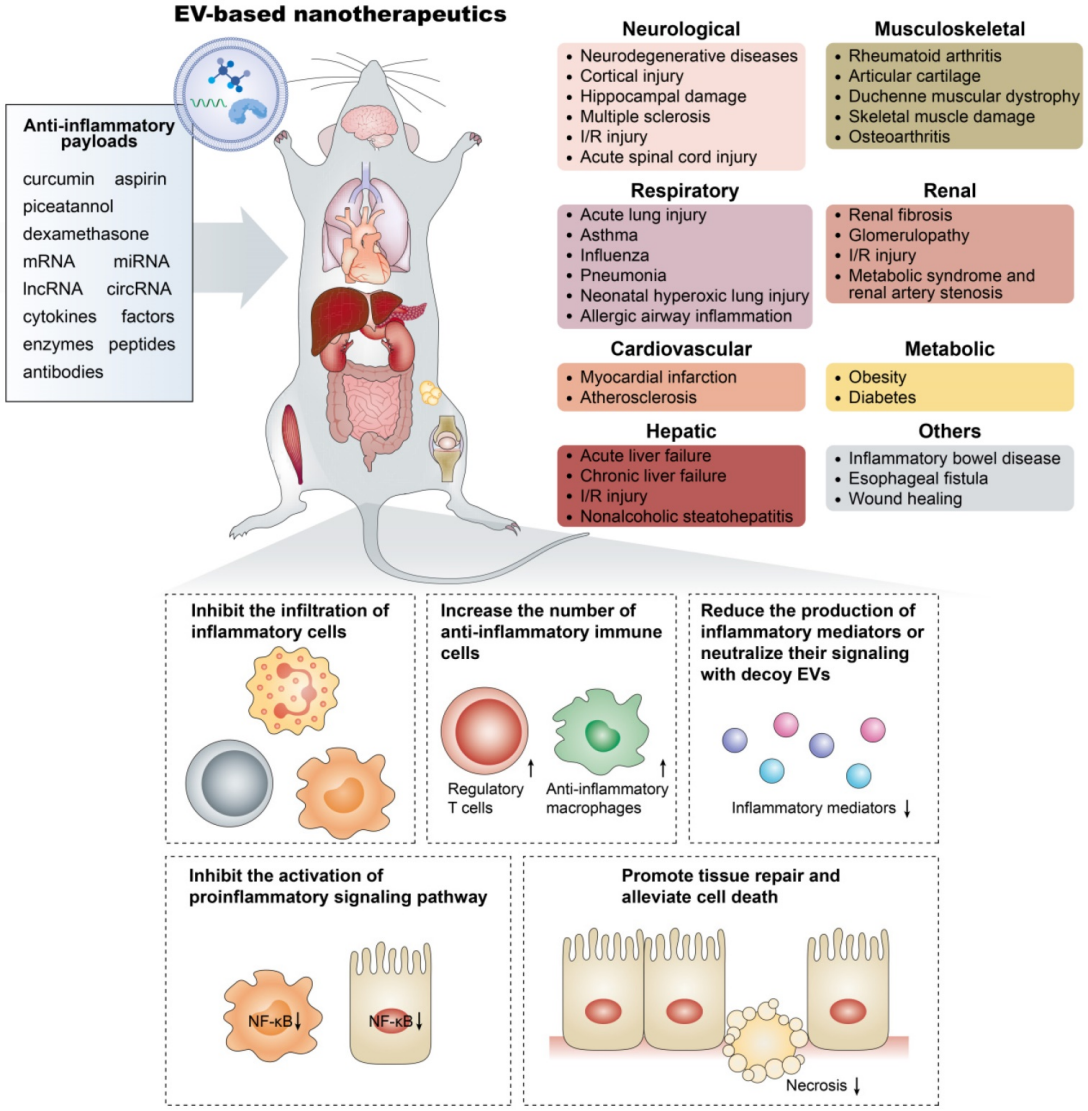
** Emerging EV-based nanotherapeutics in the treatment of inflammatory-related diseases.** The anti-inflammatory payloads in EV-based nanotherapeutics include small molecule drugs, RNA species, and therapeutic proteins, which show significant therapeutic efficacy in inflammatory-related diseases occurring in organs such as the brain, lung, heart, liver, and kidney through various mechanisms.

Overall, these examples underline the versatility of EV-based nanotherapeutics and their potential for addressing multiple unmet clinical demands (**Figure 3**). However, in addition to their efficacy, researchers should tend to attach great importance to the pharmacokinetic and pharmacodynamic profiles of EV-based nanotherapeutics to optimize and standardize their treatment strategies to hasten their clinical development. It should be noted that due to the length limitations, this review focuses on the anti-inflammatory applications of EVs in noncancer diseases, whereas comprehensive reviews on their roles in anticancer immunity are available elsewhere 160,161.

## Challenges and perspectives in the anti-inflammatory application of EV-based nanotherapeutics

**Figure 4:**
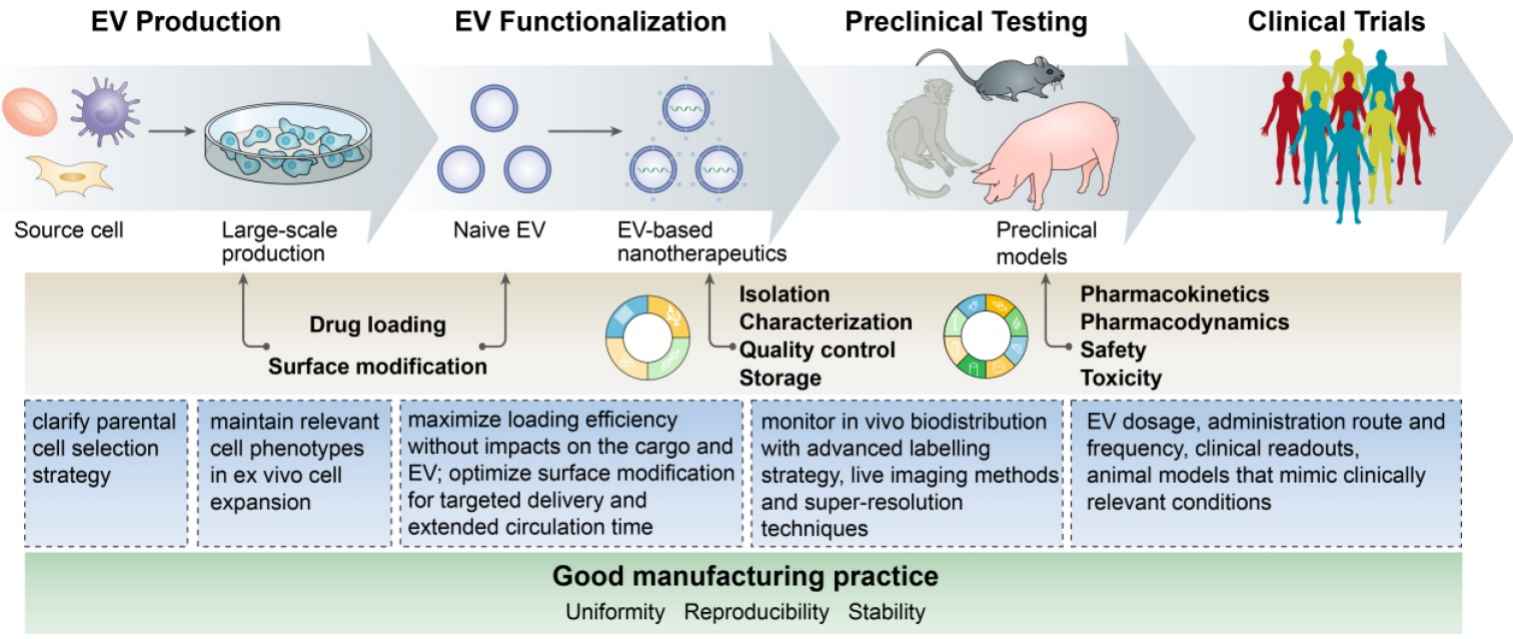
The flow and common concerns of developing EV-based nanotherapeutics.

EV-based nanotherapeutics represent a promising next-generation treatment for many inflammatory diseases by virtue of their own merits, such as desirable stability in body fluids, ability to surmount biological barriers, low immunogenicity when using autologously derived EVs, fitness for the delivery of various therapeutic agents, and ability to achieve disease-specific tailor-made delivery, which can provide many dramatic improvements to an army of drugs to fight excessive or persistent inflammation. However, a set of common concerns in the process of EV production, functionalization, and application should be ironed out before turning them into the clinic (**Figure 4**):

- **Source cell selection**: Almost all cell types can secrete EVs, but which source is suitable for EV production for anti-inflammatory applications remains unclear. Autologous primary cell-derived EVs have a low risk of immunological rejection, but suffer from lower yield, whereas cell lines can be cultured indefinitely but may carry some risk of oncogenic potential. Despite success in developing immune cell-derived EV-based delivery system, however, it should be noted that these EVs may also have the potential to provoke inflammation. How the EV source affects therapeutic efficacy and immune response needs further investigation.
- **Unwanted biological effects of the inherent cargos in EVs**: The capacity of EVs for the intracellular transmission of bioactive molecules influences the physiological and pathological functions of recipient cells. For example, heat shock proteins present on EVs may trigger unintentional immune cell activation, and consequently impact therapeutic efficacy, which deserves serious consideration when designing EV-based therapeutics. An important question is how to selectively dislodge undesirable substances from EVs during biogenesis.
- **Large-scale production**: Developing EV-based nanotherapeutics requires a production method that assures not only high quality but also high quantity. However, existing methods cannot, as yet, fully meet the standards for clinical translation. Emerging cell culture equipment, such as a hollow-fiber culture system, has been generated for the scalable and continuous production of therapeutic EVs, affording 40-fold more EVs per volume of conditioned medium than conventional cell culture 162. Moreover, physical and mechanical stress, such as sonication, extrusion and centrifugal force, have been utilized to yield EV-like cell-derived vesicles, increasing EV production by more than 100-fold compared to naturally secreted EVs 40,163,164. However, these EV mimetics may not necessarily share all common features with biogenic EVs, and whether this impacts delivery, biocompatibility, immunogenicity, or other EV properties will require stringent assessment.
- **Optimal cargo loading**: There is no doubt that the loading efficiency of EVs for different types of cargos largely determines the success or failure of EV-based therapy. Excessively rough loading approaches may ruin the EV integrity, induce immunogenicity, or lead to drug degradation and EV aggregation. Procedures that maximize the loading efficiency without compromising the stability of the cargo or the EVs themselves should be defined. For the endogenous loading, the mechanisms that mediate the sorting of cargos into EVs remain to be fully unraveled.
- **Nonspecific uptake by the mononuclear phagocyte system**: The major limitation of EV delivery is its inability to reach disease sites owing to nonspecific uptake by the mononuclear phagocyte system. Recently, Wan et al. reported that prior blockade of the mononuclear phagocyte system with siCltc-modified EVs significantly improved the protective effect of the miRNA-21-loaded EVs in doxorubicin-induced myocardial toxicity 165, suggesting that the two-step EV delivery strategy (blocking the uptake of EVs before the delivery of therapeutic EVs) would be a promising method for the treatment of heart diseases.

- **Monitoring the *in vivo* biodistribution**: Labeling strategies such as fluorescent dyes and luciferase have been used for the *in vivo* tracing of EVs 166. Direct labeling with fluorescent dyes is easy but may affect the biological behavior of the EVs. Leftover dyes could be detected rather than vesicles and may increase the risk of tissue contamination. In addition, current techniques cannot measure the precise amount of EVs in specific organs due to the thickness of the tissue and the resulting loss of signal. Thus, advances in labeling strategies, *in vivo* imaging techniques and super-resolution construction will surely aid in the dynamic tracking of EVs and their intracellular fates.
- ***In vivo* assays and safety challenges**: Although trials are already investigating the clinical potential of EV-based therapy, there is still a lack of the application note regarding many factors, such as EV dosage, administration route and frequency, and clinical readouts. Animal models that mimic clinically relevant conditions are urgently needed to evaluate the safety, toxicity and pharmacokinetics of EV-based nanotherapeutics to support clinical application.

**Figure 5:**
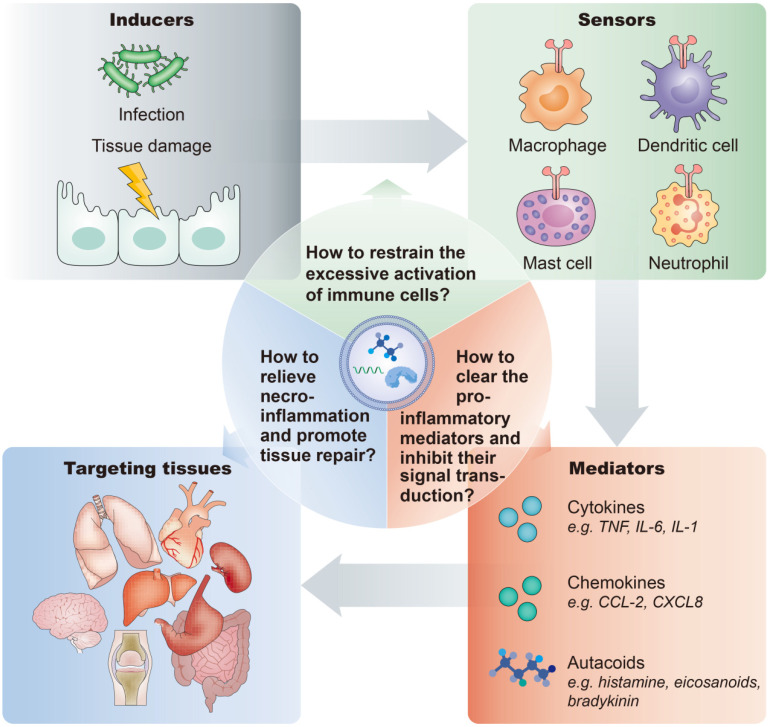
Inflammatory pathway components and key considerations of anti-inflammatory treatment. A typical inflammatory pathway consists of inducers, sensors, mediators and effectors. For the anti-inflammatory therapy, important questions are: How to restrain the excessive activation of immune cells? How to clear the pro-inflammatory mediators and inhibit their signal transduction? How to inhibit necroinflammation and promote tissue repair?

Moreover, to broaden the application of EVs in the inflammatory diseases, several issues need to be considered:

- First, the inflammatory response is orchestrated by various modulators and pathways. In general, a typical inflammatory pathway consists of inducers, sensors, mediators and effectors. Exogenous or endogenous inducers of inflammation initiate and activate the sensors, such as pattern recognition receptors expressed on immune cells, which instigate the production and secretion of various inflammatory mediators, such as cytokines, chemokines, and autacoids, thereby affecting the functions of target organs (**Figure 5**). Thus, developing rational targeting or therapeutic strategies tailored to these different stages will advance the application of EV-based nanotherapeutics for multiple inflammatory-related diseases.
- Second, it is important to take advantage of natural EVs with innate therapeutic benefit. For example, stem cell-derived EVs exhibit versatile functions, including anti-apoptosis, anti-inflammation, and anti-fibrosis activities. Exploiting these EVs as delivery vectors for anti-inflammatory agents may provide synergy and facilitate the treatment effect. Meanwhile, they may also offset the side effects of packaged drugs, such as aspirin, which can markedly inhibit inflammation but induce apoptosis.
- Additionally, the application of EVs in the anti-inflammatory therapy is still in the stage of preliminary development, and further exploitation and utilization are needed to maximize their advantages to develop theranostic platforms for anti-inflammatory therapy. For example, EVs in the circulation and body fluid can provide diagnostic information and aid in making therapeutic decisions for inflammatory disorders. EVs combined with imaging modalities will enable the visualization of inflamed tissues, enabling noninvasive monitoring of the *in vivo* efficacy of an anti-inflammatory drug.

## Conclusions

The past few decades have provided a wealth of information on how maladaptive and excessive inflammation drives a number of diseases. However, translating this knowledge to improve clinical therapy is confronted with many challenges. The field of EV-based nanotherapeutics is rapidly evolving and expanding, opening up vast opportunities for a new generation of anti-inflammatory medicines. Two overall applications are emerging: (i) exploiting the intrinsic therapeutic activity of EVs for inflammation-combating; and (ii) employing EVs as nature's own delivery carrier for small molecule drugs, therapeutic RNAs and proteins delivery in combination with targeting moieties. Despite the inspiring therapeutic potential, the field is being starved of new techniques to optimize EV production and cargo loading, target single-vesicle analysis, and track EVs with powerful imaging methods. In the future, deeper and broader cooperation in areas of medicine, nanotechnology, material science, bioengineering and pharmaceutical perspectives will surely enable better use of EVs in the treatment of inflammation.

## Figures and Tables

**Table 1:** Representative MSC-derived EVs with anti-inflammatory properties

Indication	EV source and Isolation method	Therapeutic schedule	Effective molecules	Treatment outcome
*Respiratory*				
Influenza 95	Swine BM-MSC; Differential centrifugation with ultracentrifugation	80 μg/kg body weight, single dose; intratracheal	RNAs	-Reduced virus shedding and influenza virus replication -Reduced apoptosis and proinflammatory cytokines -Ameliorated influenza virus-induced acute lung injury
Neonatal hyperoxic lung injury 96	Human UC-MSC; Differential centrifugation with ultracentrifugation	20 μg, single dose; intratracheal	VEGF protein and mRNA	-Suppressed macrophage activation and proinflammatory cytokines secretion -Improved alveolarization and angiogenesis -Attenuated hyperoxic lung injuries
Pneumonia 97	Human BM-MSC; Differential centrifugation with ultracentrifugation	dosed by total cell count, single dose; intratracheal or intravenous	KGF mRNA	-Reduced inflammatory cell infiltration and cytokines secretion -Increased bacterial clearance -Prolonged survival
Allergic airway inflammation 98	Human iPSC-MSCs; Anion-exchange chromatography	2×10^10^ particles, single dose; intravenous	miR-146a-5p	-Inhibited the function of human ILC2s *in vitro* -Alleviated ILC2 levels, inflammatory cell infiltration and mucus production in the lung, and improved airway hyperresponsiveness
*Cardiovascular*				
Myocardial I/R injury 99	Mouse BM-MSC; Differential centrifugation with ultracentrifugation	20 μg, single dose; myocardial injection	miR-182	-Converted pro-inflammatory macrophages to M2-like phenotype -Reduced infarct size and alleviated inflammation
Myocardial infarction 100	BM-MSC; ExoQuick-TC (System Bioscience)	80 μg, single dose; myocardial injection	Not studied	-Promoted angiogenesis and inhibited proliferation of lymphocytes *in vitro* -Reduced infarct size, and preserved cardiac systolic and diastolic performance
Sepsis-induced cardiac injury 101	Mouse BM-MSC; Differential centrifugation with ultracentrifugation	2 μg/g body weight, single dose; intravenous	miR-223	-Reduced polymicrobial sepsis triggered cardiac dysfunction, apoptosis and inflammatory response
*Hepatic*				
Hepatic I/R injury 102	Human UC-MSC; Differential centrifugation with ultracentrifugation	10 mg/kg body weight, single dose; intravenous	MnSOD	-Alleviated neutrophil infiltration and oxidative stress -Protected against hepatic apoptosis and restored liver function
Chronic liver failure 103	Human ESC-MSCs; Differential centrifugation with ultracentrifugation	350 μg, single dose; intraperitoneal	Not studied	-Hydrogel-mediated delivery improved the anti-fibrosis, anti-inflammation, anti-apoptosis, and regenerative effects of MSC-EVs
Acute liver failure 104	Human and mouse BM-MSC; Differential centrifugation with ultracentrifugation	2×10^8^~2×10^10^ particles, single dose; intravenous/ intraperitoneal	Y-RNA-1	-Reduced hepatic injury, modulated cytokine expression, and increased survival by systemic administration
*Renal*				
Renal I/R injury 105	Mouse BM-MSC; Differential centrifugation with ultracentrifugation	80 μg, single dose; renal capsule injection	Not studied	-Hydrogel-mediated delivery enhanced the anti-apoptosis and anti-inflammatory effects of MSC-EVs -Promoted endothelial cell proliferation and angiogenesis, and inhibited chronic renal fibrosis
Metabolic syndrome and renal artery stenosis 106	Swine AD-MSC; Differential centrifugation with ultracentrifugation	1×10^10^ particles, single dose; intrarenal injection	IL-10	-Induced monocytes to differentiate into M2-like macrophages, and reduced renal inflammation -Ameliorated renal hypoxia and scarring
Renal I/R injury 107	Human UC-MSC; Differential centrifugation with ultracentrifugation	100 μg, single dose; intravenous	miR-15a miR-15b miR-16	-Suppressed CX3CL1 expression, macrophage infiltration and cell apoptosis -Improved renal function and renal fibrosis
Renal I/R injury 108	Human UC-MSC; Differential centrifugation with ultracentrifugation	100 μg, single dose; intravenous	miRNAs	-Improved tubular injury and protected renal functions by modulating NK cells
*Neurological*				
Cortical injury 109	Monkey BM-MSC; Differential centrifugation with ultracentrifugation	4×10^11^ particles, two doses (Day 1 and 14 post-injury); intravenous	Not studied	-Reduced neuroinflammation and shifted microglia towards an anti-inflammatory phenotype -Recovered motor function
Hippocampal damage 110	Human BM-MSC; Differential centrifugation with ultracentrifugation	15 μg, two doses; intracardiac injection	Cytokines and factors	-Suppressed extensive inflammation, reactive astrogliosis, and increased integrity of the BBB -Rescued memory and learning deficiencies
Multiple sclerosis 111	Human BM-MSC; Differential centrifugation with ultracentrifugation	150 μg, single dose; intravenous	RNAs and proteins	-Decreased neuroinflammation and upregulated Tregs -Reduced demyelination and the clinical score of EAE mice
Acute spinal cord injury 112	Human BM-MSC; Tangential flow filtration	1×10^9^ particles, single dose; intravenous	Not studied	-Diminished inflammatory response with apparent astrocyte and microglia disorganization, and improved functional recovery
Preterm brain injury 113	Human BM-MSC; Differential centrifugation with ultracentrifugation	dosed by total cell count, two doses (3 h before and 24 h after injury); intraperitoneal	Not studied	-Prevented reactive astrogliosis and microgliosis -Reduced neuronal cell death, and restored white matter microstructure
*Musculoskeletal*				
Osteoarthritis 114	Human ESC-MSCs; Tangential flow filtration	100 μg, three doses (2, 4, and 8-weeks post-injury); intra-articular injection	Not studied	-Suppressed inflammation, apoptosis and matrix degradation -Promoted TMJ repair and regeneration
Inflammatory arthritis 115	Mouse BM-MSC; Differential centrifugation with ultracentrifugation	250 ng of Exos, 250 or 600 ng of MPs, two doses (Day 18 and post-injury); intravenous	Not studied	-Exerted an anti-inflammatory role on T and B lymphocytes -Exos were more efficient in suppressing inflammation *in vivo*
Articular cartilage 116	Human ESC-MSCs; Tangential flow filtration	100 μg, once a week for up to 12 weeks; intra-articular injection	CD73	-Induced the polarization of M2-like macrophages, and reduced pro-inflammatory cytokine production -Enhanced cellular proliferation and chondrocyte functions
Duchenne muscular dystrophy 117	Placenta-MSC; Differential centrifugation with ultracentrifugation	5×10^9^ particles, single dose; intra-muscular injection	miR-29c	-Promoted muscle differentiation *in vitro* -Decreasing inflammation and fibrosis in mdx mice
Skeletal muscle damage 118	Human AD-MSC; Differential centrifugation with ultracentrifugation	total 2×10^10^ particles: 1×10^10^ (immediately after injury), intravenous; 0.5×10^10^ (Day 1 and 2 post-injury), intra-muscular injection	neuregulin 1 protein	-Impaired inflammatory cell infiltration -Induce vascular growth and protect muscle against I/R damage
Skeletal muscle damage 119	Human AD-MSC; Differential centrifugation with ultracentrifugation	1 μg, single dose; intra-muscular injection	miRNAs	-Triggered macrophage polarization from a M1 to a M2 phenotype -Downregulated the pro-inflammatory cytokine IL-6 accompanied by the upregulation of IL-10
*Others*				
Obesity 120	Mouse AD-MSC; ExoQuick-TC (System Bioscience)	30 μg, once every 3 days for 6-8 weeks; intraperitoneal	STAT3 protein	-Polarized macrophages toward M2 expressing high levels of arginase-1 and IL-10 -Alleviated white adipose tissue inflammation, obesity, and hepatic steatosis, and improved metabolic homeostasis
Inflammatory bowel disease 121	Human BM-MSC; Differential centrifugation with ultracentrifugation	200 μg, single dose; intravenous	metallothionein-2 protein	-Downregulated inflammatory responses and maintained intestinal barrier integrity -Polarized M2b macrophages and subsequently induced IL-10 secretion
Retinal I/R injury 122	Human BM-MSC; Ultrafiltration followed by ExoQuick-TC (System Bioscience)	4×10^6^ particles, single dose; vitreous humor injection	Not studied	-Enhanced functional recovery, and decreased neuro-inflammation and apoptosis
Esophageal fistula 123	Swine AD-MSC; Differential centrifugation with ultracentrifugation	PF-127 gel containing 5.2×10^11^ particles injected through fistula internal and external orifices, respectively	Not studied	-Reduced the density of myofibroblasts -Decreased fibrosis and inflammatory response -Increased angiogenesis.
Burn 124	Human UC-MSC; PureExo^®^ Exosome Isolation Kit (101Bio)	800 μg (RNA concentration), single dose; intravenous	miR-181c	-Reduced burn-induced inflammation by downregulating the TLR4 signaling pathway

AD, adipose tissue; BM, bone marrow; UC, umbilical cord; iPSC, induced pluripotent stem cell; ESC, embryonic stem cell; ILC2, Group 2 innate lymphoid cell; I/R, ischemia/reperfusion; EAE, experimental autoimmune encephalomyelitis; TMJ, temporomandibular joint; Exo, exosome; MP, microparticle; VEGF, vascular endothelial growth factor; KGF, keratinocyte growth factor; MnSOD, manganese *Superoxide dismutase.*

## Abbreviations

EVs: extracellular vesicles
PEG: polyethylene glycol
ICAM-1: intercellular cell adhesion molecule-1
VCAM-1: vascular cell adhesion molecule-1
RVG: rabies viral glycoprotein
RGD: Arg-Gly-Asp
RGE: neuropilin-1-targeted peptide (RGERPPR)
EXPLORs: exosomes for protein loading via optically reversible protein-protein interactions
RNAi: RNA interference
EXOtic devices: EXOsomal transfer into cells devices
MSC: mesenchymal stem cells
IL-1RA: interleukin-1 receptor antagonist
ARDS: acute respiratory distress syndrome
CVD: cardiovascular disease
UUO: unilateral ureteral occlusion
